# Expanding Role of T Cells in Human Autoimmune Diseases of the Central Nervous System

**DOI:** 10.3389/fimmu.2017.00652

**Published:** 2017-06-07

**Authors:** Deepti Pilli, Alicia Zou, Fiona Tea, Russell C. Dale, Fabienne Brilot

**Affiliations:** ^1^Brain Autoimmunity Group, Institute for Neuroscience and Muscle Research, The Kids Research Institute at The Children’s Hospital at Westmead, University of Sydney, Sydney, NSW, Australia; ^2^Brain and Mind Centre, University of Sydney, Sydney, NSW, Australia

**Keywords:** autoreactive T cells, central nervous system autoimmune diseases, neuroimmunology, autoantibodies, multiple sclerosis, T cell detection

## Abstract

It is being increasingly recognized that a dysregulation of the immune system plays a vital role in neurological disorders and shapes the treatment of the disease. Aberrant T cell responses, in particular, are key in driving autoimmunity and have been traditionally associated with multiple sclerosis. Yet, it is evident that there are other neurological diseases in which autoreactive T cells have an active role in pathogenesis. In this review, we report on the recent progress in profiling and assessing the functionality of autoreactive T cells in central nervous system (CNS) autoimmune disorders that are currently postulated to be primarily T cell driven. We also explore the autoreactive T cell response in a recently emerging group of syndromes characterized by autoantibodies against neuronal cell-surface proteins. Common methodology implemented in T cell biology is further considered as it is an important determinant in their detection and characterization. An improved understanding of the contribution of autoreactive T cells expands our knowledge of the autoimmune response in CNS disorders and can offer novel methods of therapeutic intervention.

## Introduction

Autoimmunity is believed to be the underlying cause in a growing number of neurological disorders. Although the precise mechanisms that trigger autoimmunity have not been fully elucidated, it is known that a dysregulation in T cells is a key component, given their constitutive role in immunosurveillance (1). The archetypal neurological disease mediated primarily by T cells is multiple sclerosis (MS) (2, 3). It has been studied extensively for many years in both humans and animal models, and an informed understanding of MS has laid the groundwork for further studies in other suspected autoimmune neurological disorders. In particular, Rasmussen’s encephalitis (RE) (4) and a spectrum of paraneoplastic syndromes (5, 6) are hypothesized to be T cell driven. In other disorders, like amytrophic lateral sclerosis (ALS), T cells may conversely play a neuroprotective role (7). In addition to a dysfunctional cellular immunity, effector molecules of humoral immunity, such as autoantibodies, may concomitantly participate in autoimmunity. Although paraneoplastic syndromes have been associated with specific autoantibodies, the search for autoantibodies in autoimmune central nervous system (CNS) diseases such as MS, RE, and ALS is still ongoing.

Indeed, in recent years, a growing number of autoantibodies targeting neuronal receptors or synaptic proteins of the CNS are proving to be useful biomarkers of various neurological diseases treatable with immunotherapy (8–15). This has spurred intensive investigations to understand the mechanisms behind autoantibody responses, with emerging evidence suggesting a pathogenic role. However, it is well established that the production and sustenance of immunoglobulin-G (IgG) autoantibodies and autoantibody-producing B cells necessitates the involvement of T cells reactive against a shared protein antigen (16–19). Although this aspect of adaptive immunity has been explored less thoroughly in autoantibody-associated neuroimmune disorders, this premise has broadened studies to focus on cellular responses in the following autoantibody-associated diseases: neuromyelitis optica (NMO), acute disseminated encephalomyelitis (ADEM), stiff person syndrome (SPS), and anti-N-methyl-d-aspartate receptor (anti-NMDAR) encephalitis.

In this review, we explore the accumulating evidence of cellular immune responses in various disorders of the CNS that are predominantly T cell-driven, as well as the more newly classified group of autoantibody-associated syndromes. In particular, we focus on findings in humans, as many studies conducted in animal models are reviewed elsewhere or have been recently reviewed (1, 20–23). Common methods implemented in the study of T cell biology are also evaluated.

## Immunosurveillance of T Cells in the CNS

The CNS has been traditionally viewed as an immune privilege site that is inaccessible to T cells and other immune cells. However, it is now well recognized that T cells actively survey the CNS in the healthy state to ensure host defense against infections. Central and effector memory T cells constantly patrol the brain and spinal cord for pathogens *via* the cerebrospinal fluid (CSF) that bathes these structures (24–26). In fact, around 80% of immune cells in the CSF are T cells (27). As they travel through the subarachnoid space between the meninges, T cells interact with resident antigen-presenting cells (APCs) to sample antigens, including parenchyma-derived antigens in the interstitial fluid that drains into the CSF (28). Memory T cells can then be restimulated upon recognition of a pathogen as part of the host response.

Moreover, recent evidence confirms the presence of lymphatic vessels within the meninges of healthy mice that resemble traditional lymphatic vessels found in the periphery, both structurally and functionally (29, 30). These meningeal vessels line the dural sinuses and drain cells and fluid of the subarachnoid space directly into the deep cervical lymph node. Notably, T cells were identified in these meningeal lymphatics (30), indicating a travel route between the CNS and lymph nodes in the steady state. Together, this refutes previous notions that immune cell entry into the CNS was restricted by the apparent absence of lymphatic drainage, and further supports the concept of immunosurveillance by T cells in the CNS.

Although the blood–brain barrier (BBB) and blood–CSF barrier shielding the CNS were seen to be another mechanism exempting the CNS from immune monitoring, various adhesion molecules on their surface enable T cell migration. Egress from the blood to the CSF is dependent on the expression of P-selectin in choroid plexus stroma vessels and meningeal vessels (24, 31). In addition, the interaction of α4β1 integrin with vascular cell adhesion molecule 1 (VCAM1) on endothelial cells of the BBB is important in facilitating T cell movement into the perivascular space, as evidenced by the efficacy of natalizumab in reducing inflammation in MS (32–34).

As will be discussed later, the importance of immunosurveillance in maintaining homeostasis in the CNS is particularly evident when it is disrupted by immunosuppression. Under immunosuppression, the mobilization of immune cells into the CNS is hindered, making the body more susceptible to opportunistic infections by agents such as, JC polyoma virus (JCV), herpes simplex virus, toxoplasmosis, and *Cryptococcus* (35–37). With the host immune response dampened and the CNS unguarded, the pathogenic response goes unchecked, leading to potentially fatal diseases.

## T Cell-Mediated CNS Diseases

### Multiple Sclerosis

Multiple sclerosis is a common chronic inflammatory disease of the CNS resulting in the demyelination of neurons. Damage to the myelin sheath surrounding neuronal axons leads to the progressive loss of neurological function and affects over two million people globally (38, 39). The majority of MS patients (85%) experience a relapsing-remitting disease course (40), who can transition into a secondary progressive disease form after approximately 10 years of primary disease (41, 42). The remaining 15% of patients follow a primary progressive disease course characterized by a steady decline in neurological function from the initial attack (38). Lesions, or plaques, are traditionally thought to present in the white matter of the brain and spinal cord. However, recent studies have shown gray matter lesions to accrue through the MS disease course and dominate in progressive disease (43–49).

The current consensus argues in favor of MS as an autoimmune disease mediated by self-reactive, myelin-specific T cells (1, 38, 46), with additional components of genetic susceptibility and environmental factors (41, 50). In terms of genetic susceptibility, MS has been strongly associated with different HLA class II haplotypes, including HLA-DR15 and HLA-DQ6, although their contributions to clinical disease have yet to be uncovered (51–53). It is hypothesized that these MHC molecules are able to present target autoantigens to autoreactive components of the adaptive immune system (54, 55). Several immune system genes have also been implicated in MS disease susceptibility, including those that code for IL-17 and IL-2 receptor (51, 52).

However, genetics only partially contribute to the risk of MS disease development. A significant proportion of disease risk can be directly correlated with various lifestyle and environmental factors including vitamin D deficiency, Epstein-Barr virus (EBV) infection, and smoking (56, 57). Epidemiological studies show that there is a strong association between MS prevalence and the angle of latitude. This trend may be attributed to exposure to solar radiation and vitamin D, with vitamin D-deficient individuals more prone to developing disease (58, 59). Several studies have also shown that MS patients with lower serum levels of vitamin D to be more susceptible to relapses (60–63). Additionally, EBV infection has been strongly linked to MS disease initiation (64). While up to 95% of the general population is seropositive for the virus by early adulthood, the risk of MS will be 15 fold greater in the seropositive population than the seronegative cohort (65). Certain sequences of EBV have been hypothesized to share homology with components of the CNS, suggesting that MS autoimmunity may be initiated by molecular mimicry (66, 67).

The MS autoimmune hypothesis is supported by the predominant presence of activated T cells in active plaques (41, 50, 65, 68–70). Myelin-specific T cells are first activated in the peripheral compartment, after which they cross the BBB into the CNS as they gain the expression of the appropriate adhesion molecules and homing receptors (55, 71). Once inside the CNS, T cells are then reactivated by CNS autoantigens presented by CNS-resident APCs, contributing to the clinical disease and demyelination (72, 73).

CD4^+^ T cells have been the focus of MS autoimmunity for decades, as MHC class II-restricted T cells are preferentially activated by EAE disease induction (38). Self-reactive CD4^+^ T cells have been shown to recognize proteins of the myelin sheath, including myelin basic protein (MBP) (74, 75), myelin-associated glycoprotein (MAG) (76, 77), and myelin oligodendrocyte glycoprotein (MOG) (78) in both MS patients and healthy donors (69). However, CD4^+^ T cells in MS patients display an activated or memory phenotype with increased avidity to myelin proteins, compared to naive myelin-specific CD4^+^ T cells isolated from controls (79–82). Previously, myelin-specific CD4^+^ T cells in MS were thought to contribute to Th1-mediated inflammation, in contrast to the Th2-mediated response of myelin-reactive T cells isolated from healthy donors (83, 84). However, recent studies have demonstrated the importance of IL-23 in MS. IL-23 is necessary for the regulation of the proinflammatory IL-17-secreting Th17 cell lineage, which have been described as the pathogenic mediators of several autoimmune diseases (85–87). This is supported by the upregulation of IL-17 gene expression in the brain lesions of MS patients as measured by microarray analysis (88, 89). The levels of Th17 cells in the CSF of relapsing MS patients were elevated in comparison to non-inflammatory neurological disease controls, whereas there were no differences in the percentages of IFN-γ-secreting Th1 cells (90). Th17 cells were also raised in the peripheral compartments of MS patients during relapses, implicating their possible relevance to disease activity (91, 92). Th17 cells were additionally demonstrated to home to active regions of lesions and areas of inflammatory demyelination, and are a major constituent of perivascular cuffs (93). The high expression of granzyme B by myelin-specific Th17 cells also promotes the death of human neurons (94). These works combined strongly insinuates Th17 cells as a potential mediator of MS pathogenesis.

The focus of research in MS has recently shifted from a predominantly CD4^+^ T cell field to include CD8^+^ cytotoxic T cells as a novel effector cell type in MS pathology (95, 96). CD8^+^ T cells have been shown to outnumber CD4^+^ T cells in MS plaques up to 10-fold at all stages of disease progression (47, 97–100). Oligoclonal expansion within the CD8^+^ T cell compartment is elevated compared to CD4^+^ T cells in lesions, CSF, and peripheral blood of MS patients (95, 96, 101). In contrast to its constitutive low expression in the CNS, MHC class I is highly upregulated on neurons and glial cells within MS lesions, which proposes that CD8^+^ T cells may be interacting with these cells (100, 102, 103). A significant number of activated or memory CD8^+^ T cells are capable of secreting the proinflammatory cytokine, IL-17, similar to the Th17 cells mentioned earlier (93). Histological analysis of MS lesions has also revealed that granzyme B-positive CD8^+^ T cells are often located adjacent to regions of demyelination (47, 104, 105). Expectedly, the levels of CD8^+^ T cells within lesions have been positively correlated with the magnitude of axonal injury (106). These findings encourage the hypothesis that CD8^+^ T cells play a role in the demyelination of axons in MS lesions.

The monoclonal antibody natalizumab is successfully used as an immunosuppressant in diseases such as MS. Natalizumab therapy is administered to MS patients who are unresponsive to first-line immunotherapies, as well as those with severe clinical disease (107). It has shown a 68% reduction in the annualized relapse rate of MS patients and has decreased the probability of sustained disability progression by 42% over the course of 2 years (108). Natalizumab targets the α4β1 integrin on T cells, thereby preventing T cells binding to VCAM1 on endothelial cells of the BBB and subsequent egress into the CNS. This is evidenced by the significant decline of several populations of T cells in the CSF of natalizumab-treated MS patients compared to controls (34). As such, immune surveillance within the CNS is compromised, which can lead to various inherited or acquired immune deficiencies (37). In particular, the use of natalizumab has been associated with opportunistic infections, most significantly JCV infection or reactivation leading to potentially lethal progressive multifocal leukoencephalopathy (inflammation of white matter in the brain) in 4 out of 1,000 treated patients (37).

Based on a review of the literature, it seems apparent that MS is a multifaceted autoimmune disease with potential contributions from Th17 cells and CD8^+^ T cells in demyelination (Table 1). Although T cell dependency is well established, the quest for potential autoantibodies in MS is still going strong (109). Popularly studied autoantigens in this field include MOG and aquaporin 4 (AQP4), although extensive research into these targets reveal that they are not, in fact, associated with MS (11, 110).

**Table 1:** Summary of findings of T cell activity in T cell-associated central nervous system diseases.

Disease	T cell antigen	Implicated T cell subset(s) and dysregulation of associated cytokines, chemokines, and other inflammatory mediators	HLA associations	Associated antibody	Reference
Multiple sclerosis	MBP, MAG, MOG	Th17: IL-23, IL-17, granzyme B CD8^+^ T cells: IL-17, granzyme B	HLA-DR15, HLA-DQ-6	Unknown	Andersson et al. (76), Cua et al. (85), Hafler et al. (51), International Multiple Sclerosis Genetics et al. (52), Montes et al. (88), Olsson and Hillert (53), Pette et al. (74), Raine et al. (89), Tsuchida et al. (77), Valli et al. (75), Zhang et al. (78)

Rasmussen’s encephalitis	Unknown	CD8^+^ T cells: granzyme B Unknown source: IL-6, TNF-α, IFN-γ	HLA-DR6 (possible)	Unknown	Andermann et al. (111), Bien et al. (112), Takahashi et al. (113), Tekgul et al. (114)

Paraneoplastic syndromes	Hu, Ma2, Yo	CD8^+^ T cells: granzyme B Unknown source: IFN-α, IL-12	Hu: HLA-DR3, HLA-DQ2 Yo:HLA-A24	Hu, Ma2, Yo, CRMP5/CV2, amphiphysin	Benyahia et al. (115), Darnell et al. (116), De Graaf et al. (117), Domschke et al. (118), Leypoldt and Wandinger (119), Rousseau et al. (120), Tanaka et al. (121), Tanaka and Tanaka (122)

### Rasmussen’s Encephalitis

Rasmussen’s encephalitis is a chronic pediatric inflammatory neurological disorder characterized by drug-resistant focal seizures, unihemispheric inflammation and atrophy, and unilateral movement disorders accompanied by progressive neurological decline (112, 123, 124). Lymphocytic and microglial nodules are commonly observed upon histopathological analysis of RE brain specimens, along with perivascular cuffing of infiltrating T cells, neuron and astrocyte death, and gliosis of the diseased hemisphere (4, 124, 125). RE has not yet been associated with any disease-specific autoantibodies, and the presence of autoantibodies to glutamate receptor 3 is secondary to and not causative of disease (124, 126–129). In fact, RE has been hypothesized to be a T cell-mediated disease based on the dominant influx of CD8^+^ T cells into active brain lesions at the initiation of disease (4) (Table 1). 7% of these infiltrating CD8^+^ cells are granzyme B-positive, and vesicles were often found positioned adjacent to MHC class I-expressing neurons and astrocytes with their granules polarized toward their target(s), suggesting a cytotoxic T cell-mediated disease course of RE (112). In addition to increased levels of granzyme B at initial stages of disease (113), Tekgul et al. revealed raised concentrations of the cytokine IL-6 in the CSF of RE patients compared to controls (114). A correlation was then established between the magnitude of neuronal death and inflammation with the level of IL-6 in the CNS of these patients, based on magnetic resonance spectroscopy (114). The overproduction of IL-6 has been attributed to overstimulation of TNF-α early in disease (113). Takahashi et al. have also shown that excessive IFN-γ production during the early stages of disease induces the secretion of IL-12 from macrophages (113).

Analysis of the T cell receptor (TCR) repertoire in the CNS and periphery of RE patients revealed clonal expansions of CD8^+^ T cells in both compartments, suggesting the presence of an antigen-specific T cell response (130). This is in contrast to normal TCR distribution in stroke patient controls (130). The number of peripheral CD8^+^ T cell clones has also been shown to correlate with the magnitude of unihemispheric atrophy (131). Although the disease epitope for RE has not yet been elucidated, the identification of a CD8^+^ T cell-mediated response in this disease expands potential treatment options, as seizures may be refractory and poorly responsive to anti-epileptic drugs (124). As a result, some patients may require invasive procedures such as hemispherectomy to regulate seizure frequency (124). Novel T cell-specific immunotherapies, like T cell blockade from the CNS with natalizumab, are therefore a promising alternative (124, 132, 133).

### Paraneoplastic Syndromes

Diseases in which the body’s immune system is altered in response to cancer are termed paraneoplastic syndromes. When paraneoplastic syndromes disturb the CNS, the effects can be far more severe than the initiating tumor, with significant disability taking hold over short periods of time (134). In CNS paraneoplastic syndromes, paraneoplastic antibodies are present at higher titers in CSF versus serum, insinuating that they are synthesized intrathecally (135). These onconeuronal IgG antibodies target intracellular neuronal antigens expressed ectopically by the tumor (136). Paraneoplastic antibodies are important biomarkers of disease, but appear unrelated to pathogenesis (137). Instead, pathogenesis may be mediated by T cells targeting the same autoantigens as the onconeural antibodies present (5) (Table 1). This hypothesis is supported by the presence of disease-specific T cells in the peripheral blood and CSF of patients with anti-Yo (cdr2) (116) and anti-Hu antibodies (115, 120). Extensive T cell infiltration into the CNS in patients with anti-Ma2 (138) and anti-Hu antibody-associated paraneoplastic encephalitis (139) has also been observed, and along with poor responses to humoral immunotherapies (140–142), supports a T cell-mediated pathogenesis of CNS paraneoplastic syndromes.

CD8^+^ cytotoxic T cells have been implicated in paraneoplastic limbic encephalitis and are associated with autoantibodies against intracellular antigens, mainly Hu (139) and Ma2, as well as CRMP5/CV2 and amphiphysin (5). In comparison to encephalitides with neuronal cell-surface antigen-directed autoantibodies, T cells in anti-Ma2 and anti-Hu paraneoplastic encephalitis are preferentially skewed toward a CD8^+^ phenotype, with a significantly higher number of activated cytotoxic granzyme B-positive cells found in close proximity to injured neurons (143, 144).

There is a limited number of studies that detail the cytokine profile in paraneoplastic patients. Autoreactive T cells in paraneoplastic breast cancer patients were found in association with elevated intratumoural levels of IFN-α and IL-12, a correlation unseen in antibody-negative breast cancer patients (118). As IL-12 is concomitant with T cell activation and function, the increase of this cytokine likely promotes the expansion of autoreactive T cells in paraneoplastic syndromes (118, 145). These results collectively suggest that paraneoplastic encephalitides are mediated by cytotoxic, antigen-specific CD8^+^ T cells, in which onconeuronal antibodies may exist as an epiphenomenon.

### Amyotrophic Lateral Sclerosis

Amytrophic lateral sclerosis is a neurodegenerative disease of the motor neurons resulting in progressive muscle paralysis. The underlying mechanisms of ALS have not yet been elucidated, and therapies to modify or delay the advancement of the disease are still being trialed. Intriguingly, recent studies have shown that CD4^+^ T cells infiltrating the spinal cord in ALS patients and mice lie adjacent to degenerating motor neurons and activated microglia (7, 146–148). However, global immunosuppression does not appear to be effective in ALS treatment, suggesting that these T cells may, in fact, rescue motor neuron death (7). There have been several studies investigating the neuroprotective mechanisms of CD4^+^ T cells following injury (7, 149–152). The data collected thus far suggests that CD4^+^ T cells in ALS mediate motor neuron survival in a highly regulated process (7).

## T Cells in Antibody-Associated CNS Diseases

### Neuromyelitis Optica

The most compelling evidence for autoreactive T cell involvement in an autoantibody-associated disease comes from studies in neuromyelitis optica (Table 2). NMO is an aggressive demyelinating disease that is distinguished from MS by the presence of specific IgG1 antibodies against AQP4 (153, 154), a water channel abundantly expressed by astrocytes in the CNS. Anti-AQP4 antibodies are detected in a significant proportion (up to 75%) of NMO patients (110, 155) and have become an important diagnostic tool. However, involvement of other immune mechanisms has been theorized as several lines of evidence, while inconclusive, indicate that anti-AQP4 antibodies alone do not induce complete pathogenesis. For example, there are incongruences in NMO induction in animal models by passive transfer of anti-AQP4 IgG alone (156–158), and high titers of anti-AQP4 antibodies were detected in humans during remission (159, 160). Furthermore, B cell-targeted immunotherapies do not always ameliorate the disease (161, 162).

**Table 2:** Summary of findings of T cell activity in antibody-associated central nervous system diseases.

Disease	T cell antigen	Implicated CD4^**+**^ T cell subset(s) and dysregulation of associated cytokines and chemokines	HLA associations	Associated antibody	Reference
Neuromyelitis optica	AQP4	Th1: IFN-γ; Th17: IL-17, IL-6, IL-10	HLA-DRB1*03, HLA-DRB3, HLA-DP1*0501	Anti-AQP4 IgG	Brum et al. (163), Deschamps et al. (164), Matsuya et al. (165), Tanaka et al. (166), Uzawa et al. (167), Vaknin-Dembinsky et al. (168), Varrin-Doyer et al. (169), Wang et al. (170), Zephir et al. (171)

Acute disseminated encephalomyelitis	Unknown	Th1: IFN-γ, TNF-α, IL-2; Th2; IL-4, IL-6, G-CSF, IL-10; Th17: IL-17, IL-6, G-CSF, IL-10; Chemokines: CXCL10, CCL1, CCL7, CCL22	Unknown	Anti-MOG IgG	Dale and Morovat (172), Ichiyama et al. (173), Ishizu et al. (174), Jorens et al. (175), Pohl-Koppe et al. (176), Yoshitomi et al. (177)

Stiff person syndrome	GAD65	Th1: IFN-γ; Th2: IL-13, IL-4, IL-5	HLA-DQB*0201, HLA-DRB1*0301	Anti-GAD IgG	Costa et al. (178), Hanninen et al. (179), Hummel et al. (180), Pugliese et al. (181), Schloot et al. (182), Skorstad et al. (183)

Anti-NMDAR encephalitis	Unknown	Th1: IFN-γ, TNF-α; Th17: IL-17, IL-6, IL-23; Chemokines: CXCL10	Unknown	Anti-NMDAR IgG	Byun et al. (184), Kothur et al. (185), Lee et al. (186), Liba et al. (187), Ulusoy et al. (188)

Since the early description of CD3^+^ T cells in active NMO lesions (189), there is mounting evidence of cellular involvement in NMO. In fact, activated T cells infiltrate NMO patient-derived lesions (190) and clonal expansion of T cells was reported (191). Efforts have been made to define the immunodominant epitope by identifying which peptide from a human AQP4 (hAQP4) peptide library induced the greatest T cell proliferation when cultured with peripheral blood mononuclear cells (PBMCs) from anti-AQP4 antibody-positive NMO patients compared to MS subjects and healthy controls (165, 168, 169). However, further studies are required to precisely define the dominant target region as these epitopes differed greatly between studies. This discrepancy could be due to different populations with varied HLA associations, or different stages of the disease in which subjects were sampled. Indeed, a longitudinal analysis of NMO patients has demonstrated a change in reactivity and specificity of T cells toward the hAQP4 peptides over time (168). Relapses in the disease were associated with elevated CD69^+^ activated T cells compared to remission (165), highlighting the possible intermittent role of T cells during an NMO attack, and thus emphasizing the importance of understanding the T cell response to monitor the disease course.

Cytokine profiling helps elucidate the functional properties of AQP4-specific T cells and has revealed that these cells exhibit predominantly a Th17 bias but also a Th1 response. Compared to MS patients or healthy controls, increased secretion of IL-17, IL-10, IL-6, and IFN-γ have been reported in the CSF (166, 167), peripheral blood (168, 169, 192), and epitope-specific T cell lines derived from NMO patients (168). Secretion of IL-17 from Th17-biased AQP4-specific T cells promoted neutrophil infiltration, which was consistent with pathological findings (169, 189). In particular, elevated IL-6, a cytokine important for Th17 differentiation, may promote survival of AQP4-specific Th17 cells while suppressing FOXP3^+^ Treg function (193–195). Furthermore, tocilizumab, a monoclonal antibody against IL-6 receptor, ameliorated the disease in NMO patients unresponsive to standard immunotherapy (196, 197).

Genetics may be a determinant of autoimmunity and indeed, there appears to be a HLA haplotype association in NMO. Depending on the ethnicity of the cohort, there is an over-representation of HLA-DRB1*03, HLA-DRB3, or HLA-DPB1*0501 in anti-AQP4 antibody-positive NMO patients (163, 164, 169–171, 198). Interestingly, Varrin-Doyer et al. demonstrated that the hAQP4 epitope they identified induced the highest T cell reactivity in NMO patients that were HLA-DR carriers (169). However, there needs to be more definitive analysis as a distinct HLA allele could not be determined based on the T cell response to a different set of AQP4 epitopes (165).

While the triggers of autoimmunity remain elusive, like in many other autoimmune diseases, molecular mimicry has been implicated in the generation of AQP4-specific T cells. In addition to proposing AQP4-specific T cell epitopes, Varrin-Doyer et al. revealed a 90% homology between the immunodominant AQP4 epitope and *Clostridium perfringens* adenosine triphosphate-binding cassette transporter permease, and a 60–70% homology to other commensal and pathogenic *Clostridium* species (169). Not only could these microbes serve to display cross-reactive determinants, the *Clostridium* species may also augment a Th17-biased response as demonstrated in mice (169, 199). Nevertheless, further investigations into molecular mimicry are required to ascertain the extent of its contribution to the development of AQP4-specific T cells.

### Acute Disseminated Encephalomyelitis

Acute disseminated encephalomyelitis is a monophasic inflammatory demyelinating disease predominantly affecting children. It can have postinfectious origins but in a subset of patients (27–47%) (200), extensive evidence implicates pathogenic autoantibodies against MOG, a protein on the outer surface of the myelin sheath (201–206). Interestingly, findings predating the discovery of anti-MOG antibodies in ADEM (205) provide support for an autoimmune T cell response.

The majority of literature supporting T cell involvement in ADEM stems indirectly from analyses of chemokines and cytokines (Table 2). Concurrent recruitment of Th1 and Th2 cells has been proposed as there was an increase in their signature chemokines, CXCL10, CCL1, CCL7, and CCL22 in the CSF of adults with ADEM compared to MS and healthy controls and was correlated with an increase in pleocytosis (207). Dysregulation in cytokine production was not distinguished in adults, but IFN-γ, TNF-α, IL-2, IL-10, IL-6, and G-CSF were upregulated in separate pediatric ADEM cohorts (172–176), further supporting the contribution of Th1 and Th2 cells. Pohl-Koppe et al. hypothesized that Th1 cells contribute to the deleterious effects of the disease, while Th2 cells predominate in the recovery of ADEM as they reported an absence of IFN-γ but an increase in IL-4 in patients during the recovery phase (176). Consistent with this, there was increased IFN-γ^+^CD3^+^ T cells in the peripheral blood during the acute stage of ADEM (177).

Conversely, as IL-6, G-CSF, and IL-10 are pleiotropic, their elevation along with IL-17A, but little Th1 and no Th2 cytokines, in the CSF of anti-MOG antibody-positive children favors a Th17 phenotype (208). Interestingly, this increase in Th17 cytokines correlated with an increase in B cell-associated cytokines and chemokines, suggesting possible interactions between multiple cell types in mediating demyelination (208). Likewise, CSF IL-6 levels correlated with the presence of plasma anti-MOG antibodies in acquired demyelinating syndromes like ADEM (209). It can then be proposed that, like in NMO, IL-6 signaling is a suitable target for treatment in anti-MOG antibody-positive patients resistant to conventional immunotherapy (196, 197). These preliminary, albeit conflicting, reports of functional helper T cells warrant investigations into autoreactive T cells themselves, but also in combination with the recent developments in anti-MOG antibodies to assess the interplay between the humoral and cellular components of the autoimmune response in ADEM (201, 204, 210).

### Stiff Person Syndrome and Other Anti-Glutamic Acid Decarboxylase Glutamic Acid Decarboxylase (GAD) Antibody-Associated Neurological Disorders

Markedly high titers of autoantibodies against glutamic acid decarboxylase (GAD) are a hallmark of non-paraneoplastic SPS and variants of cerebellar ataxia, limbic encephalitis, and epilepsy (211–214). As GAD is an enzyme involved in the synthesis of the inhibitory neurotransmitter γ–aminobutyric (GABA), the current hypothesis is that anti-GAD antibodies disrupt GABAergic signaling. Indeed, *in vitro* and *in vivo* studies demonstrate the potential pathogenicity of anti-GAD antibodies (215–217). The salient question remains, however, of the mechanism underlying autoantibody recognition of a cytoplasmic antigen like GAD, which is unlike other known extracellular antigens targeted by pathogenic autoantibodies (218).

Given the variety of anti-GAD antibody-associated neurological disorders, it is plausible that antigen-specific T cells play an additional role in pathogenesis that differentiates the diseases (Table 2). This is an important aspect for investigation but there is a paucity of studies examining cellular mechanisms despite reports of CNS infiltration of lymphocytes in these patients (219). In a study comparing SPS with cerebellar ataxia associated with polyendocrine autoimmunity (CAPA), both cohorts presented with high titers of anti-GAD antibodies (178). Yet, cell proliferation and the percentage of HLADR^+^CD3^+^ activated T cells in response to GAD65 protein was significantly greater in SPS but not CAPA. Monitoring the course of SPS revealed a constant reactivity of CD4^+^ T cells against GAD65, which notably correlated with high anti-GAD antibody titers (179). A few other groups have identified GAD65-specific T cells in the blood but these were weakly responsive to GAD65 (180, 182, 220, 221). To this end, Skorstad et al. argue that GAD65-specific T cells largely reside in the CNS along with B cells to collaborate in the intrathecal production of anti-GAD antibodies as they were more successful in identifying and cloning GAD65-specific T cells from CSF than from blood (183). Furthermore, using overlapping GAD65 peptides, putative T cell epitopes have been identified but differ between studies and depending on whether T cell lines were generated from the blood or CSF (179, 182, 183, 220). As has been demonstrated with anti-GAD65 antibody epitopes (222) [recently reviewed in Ref. (223)], differences in T cell epitopes have been shown to be a distinguishing factor between SPS and type 1 diabetes, another anti-GAD antibody-associated disease (220, 221).

Exploration of the cytokine environment to determine the phenotype of T cells has indicated largely a Th2 bias. Secretion of IL-13, IL-4, and IL-5 reported in SPS (179, 182, 183) supports a non-inflammatory environment wherein disease is driven by autoantibodies. IFN-γ production, indicative of a Th1 response, was also recorded (182, 183) and subsequently reduced upon treatment with immunotherapy, and coincided with clinical improvement (180). It was proposed that high levels of IFN-γ production occurs in the early phase of SPS but is later exceeded by significant production of IL-13, alluding to a shift from Th1 to Th2 (179). While low or undetectable in SPS, there was a notable production of IFN-γ, and hence a dominant Th1 response is observed in CAPA (178) and type 1 diabetes (220).

T cell involvement is further supported by preliminary findings on HLA allele correlations in SPS. Pugliese et al. described a strong association between SPS and carriers of HLA-DQB1*0201 haplotype (181). HLA-DRB1*0301 has additionally been proposed as a correlate of SPS but the validity of this finding is hampered by small sample size (178, 179).

### Anti-NMDAR Encephalitis

Anti-NMDAR encephalitis is the prototypic autoimmune encephalitis associated with autoantibodies against cell-surface antigens. Discovery of the specific anti-NMDAR antibody (224) has sparked considerable interest in humoral mechanisms of this disease [recently reviewed in Ref. (225)], leading to the current hypothesis that anti-NMDAR antibodies exhibit pathogenic effects *via* internalization of the surface receptor, thereby resulting in reversible NMDAR hypofunction (226–228).

To date, there are limited and small studies investigating cellular responses in anti-NMDAR encephalitis but nevertheless, prompt further exploration (Table 2). Evidence of T cell involvement derives from cytokine and chemokine profiling and mainly favors a Th17 response. Based on significantly elevated serum levels of IL-17 and IL-6 in anti-NMDAR antibody-positive patients compared to controls (184), Byun et al. hypothesized that undetected Th17 cells secrete IL-17, which promotes a positive feedback loop of IL-6 signaling that facilitates intrathecal antibody production observed in most patients (224, 229). In line with this finding, targeting the IL-6 receptor with tocilizumab in rituximab-resistant patients with suspected autoimmune encephalitis demonstrated marked improvements (186), as was also seen in NMO. Upregulation of serum IL-23 strengthens the case for Th17 activity (188). There appears to be some heterogeneity in T cell lineage as higher levels IFN-γ and TNF-α were observed in the CSF, indicative of a Th1 cytokine dysregulation (185, 187). Consistent with T cell involvement in anti-NMDAR encephalitis was the increased level of T cell-related chemokine CXCL10 in patient CSF, which correlated with CSF pleocytosis (187).

On the other hand, there is contentious evidence of T cell involvement. Immunopathological analysis of brain sections from anti-NMDAR encephalitis patients show some to no evidence of T cell infiltration in the parenchyma and perivascular space, which disqualifies CD8^+^ cytotoxic T cells as drivers of the disease (143, 230–232). However, this does not preclude the possibility of NMDAR-specific T cell involvement in B cell activation in the periphery, prior to anti-NMDAR antibodies trafficking to the CNS.

## Evaluating T Cell Detection Methods

Choice of methods is a key determinant in discovering and studying T cell biology. An important consideration is the rarity of antigen-specific T cells, especially the proportion reactive against auto-antigens which is typically less than 0.01% of the total T cell repertoire (233). It is therefore imperative that sensitive yet specific techniques are implemented when analyzing antigen-specific T cells. Widely used T cell detection methods can be broadly distinguished into two categories: techniques that identify and assess specificity and assays that examine the functionality (Table 3).

**Table 3:** Evaluation of major techniques used in the analysis of human antigen-specific T cells.

Technique	Advantages	Disadvantages
**Identification of antigen-specific T cells**		

Peptide-MHC (pMHC) multimers	Highly specific interaction between T cell receptor and its cognate antigenic peptide presented by the multimer Independent of functional status of cells Labeled T cells can be isolated and purified for further characterization	Requires prior knowledge of epitope and its HLA haplotype restriction Does not provide functional details of identified antigen-specific T cell More difficult to develop multimers for CD4^+^ T cells

Detection *via* activation markers	Independent of epitope and HLA haplotype restriction Allows characterization of all antigen-specific T cells, irrespective of subtype Identified cells are viable, allowing for isolation and purification for further characterization	Unless appropriate activation markers are selected, results may be confounded by marker expression on non-stimulated T cells and bystander activation

**Functional assays**		

[^3^H]-thymidine incorporation	Demonstrates the proliferative capacity of antigen-specific T cells Allows for detection of numerous antigen-specific T cells	Source of cytokine is not available, making it an indirect method of T cell detection Phenotype of proliferative cells cannot be determined Results may be confounded by bystander activation Frequency of T cells in original sample cannot be elucidated

Carboxyfluorescein succinimidyl ester (CFSE) dilution assay	Demonstrates the proliferative capacity of antigen-specific T cells Allows for detection of numerous antigen-specific T cells If used in conjunction with antibodies against activation markers, the phenotype of the proliferative cells may be determined	An indirect method of T cell detection if CFSE used alone Frequency of T cells in original sample may be confounded by bystander activation CFSE may interfere with normal cellular processes

Enzyme-linked immunospot (ELISPOT)	Can enumerate cells capable of secreting cytokine of interest and categorize them into likely T cell subsets Can characterize cytokine kinetics based on spot morphology Highly sensitive, even in small samples	Selection of cytokine for analysis is based on hypothesis of its relevance Restricted to analysis of maximum two cytokines per experiment Frequency of antigen-specific T cells may be underestimated due to non-functional cells and also possible secretion of cytokines other than that being tested by assay Source of cytokine is not available, making it an indirect method of T cell detection

Intracellular cytokine staining (ICS)	Allows simultaneous determination of cytokines produced and phenotype of cells producing the cytokines, if antibodies against activation markers used in conjunction Quantify cytokine produced per cell	Selection of cytokine for analysis is based on hypothesis of its relevance Requires larger sample than ELISPOT Cells not viable for further analysis due to fixation and permeabilization

**Figure 1:**
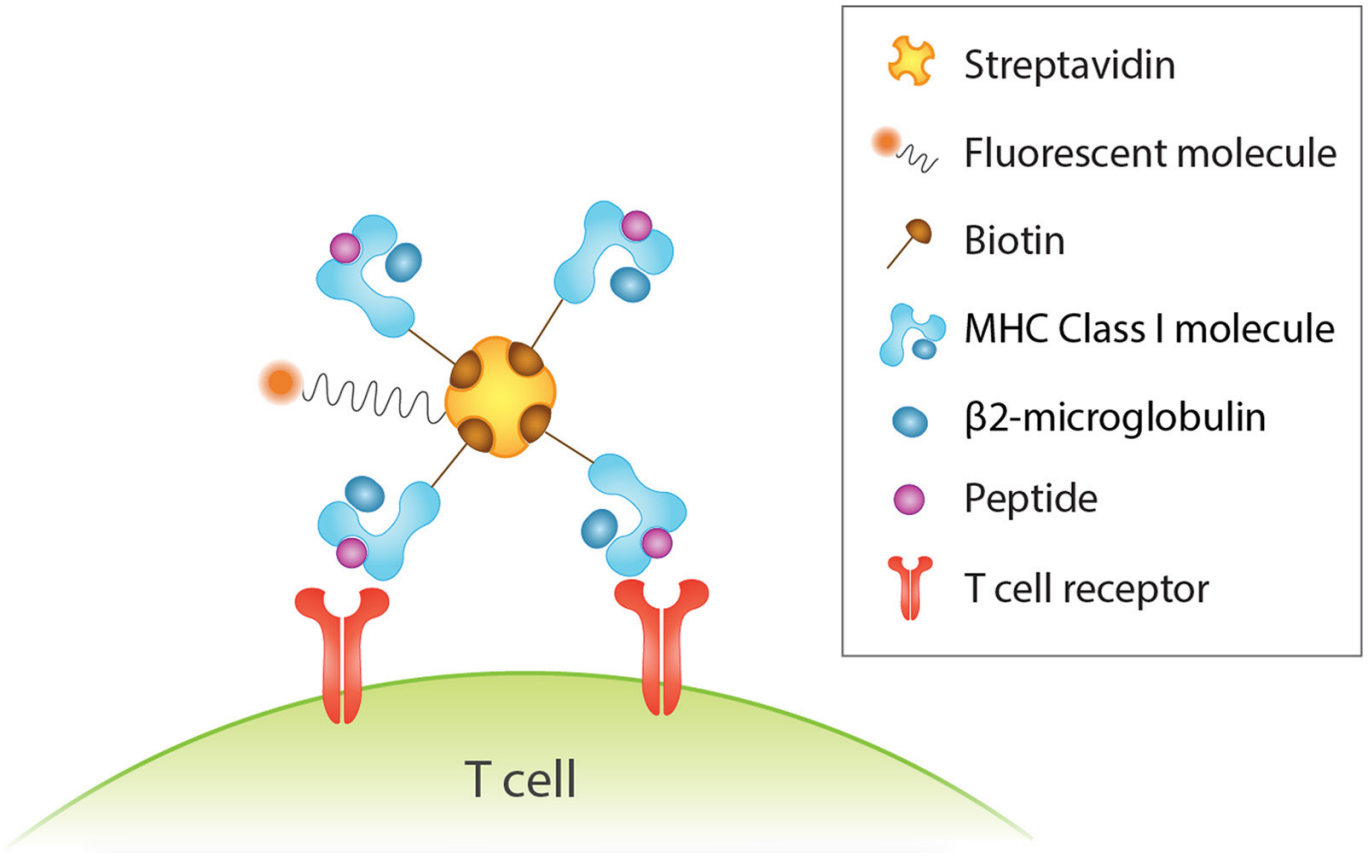
Detection of human antigen-specific T cells with peptide-MHC (pMHC) multimer. Binding four pMHC monomers, for instance, *via* biotin–streptavidin interactions increases binding avidity between antigen and T cell receptor. This in turn enhances the sensitivity and specificity of antigen-specific T cells detected by flow cytometry analysis *via* the fluorescent streptavidin.

An important tool for identification of antigen-specific T cells is peptide-MHC (pMHC) multimers. This rapidly evolving technology involves the formation of a complex of peptide-loaded MHC monomers *via* biotinylation with a fluorescently labeled streptavidin (Figure 1), which increases binding avidity (234) and overcomes the issue of low affinity binding and fast dissociation rate between TCRs and pMHC monomers (235). The value of this method lies in the direct and specific recognition and isolation of T cells *via* flow cytometry that is independent of their biological activity, such as anergic cells that are incapable of proliferation and cytokine production (236–239). However, this is a double-edged sword, as knowledge of the functional characteristics of the identified T cells allows for a deeper understanding of their response. In addition, a major drawback of this approach is that it necessitates knowledge of the T cell epitope and its MHC haplotype association (236). While pMHC multimers are extensively used for study of CD8^+^ T cells, their use in the study of MHC class II-restricted CD4^+^ T cells is challenged by the lower frequency of antigen-specific CD4^+^ T cells in the peripheral blood (240) but also largely by the difficulty in creating them because of variations in MHC structure and TCR affinity (236, 238).

Alternatively, antigen-specific T cells can be directly identified and isolated for downstream functional characterization by probing for activation markers expressed on the surface of T cells upon antigenic stimulation. A major advantage of this technique is that unlike pMHC multimers, it does not require knowledge of the antigenic epitope and associated MHC haplotypes (236) and is effective for studying CD4^+^ T cells. A multitude of activation markers have been proposed, including CD25, CD69, CD40L, CD134, CD137, and HLA-DR (241–245). Such markers are favorable indicators of an antigenic-specific response as their surface expression is contingent on activation, but absent or minimally expressed in the resting phase, and occurs for a transient period of time. Moreover, like pMHC multimer staining, expression of some activation markers is irrespective of T cell function and its differentiation state (236, 244), allowing for an unbiased characterization of antigen-specific T cells.

**Figure 2:**
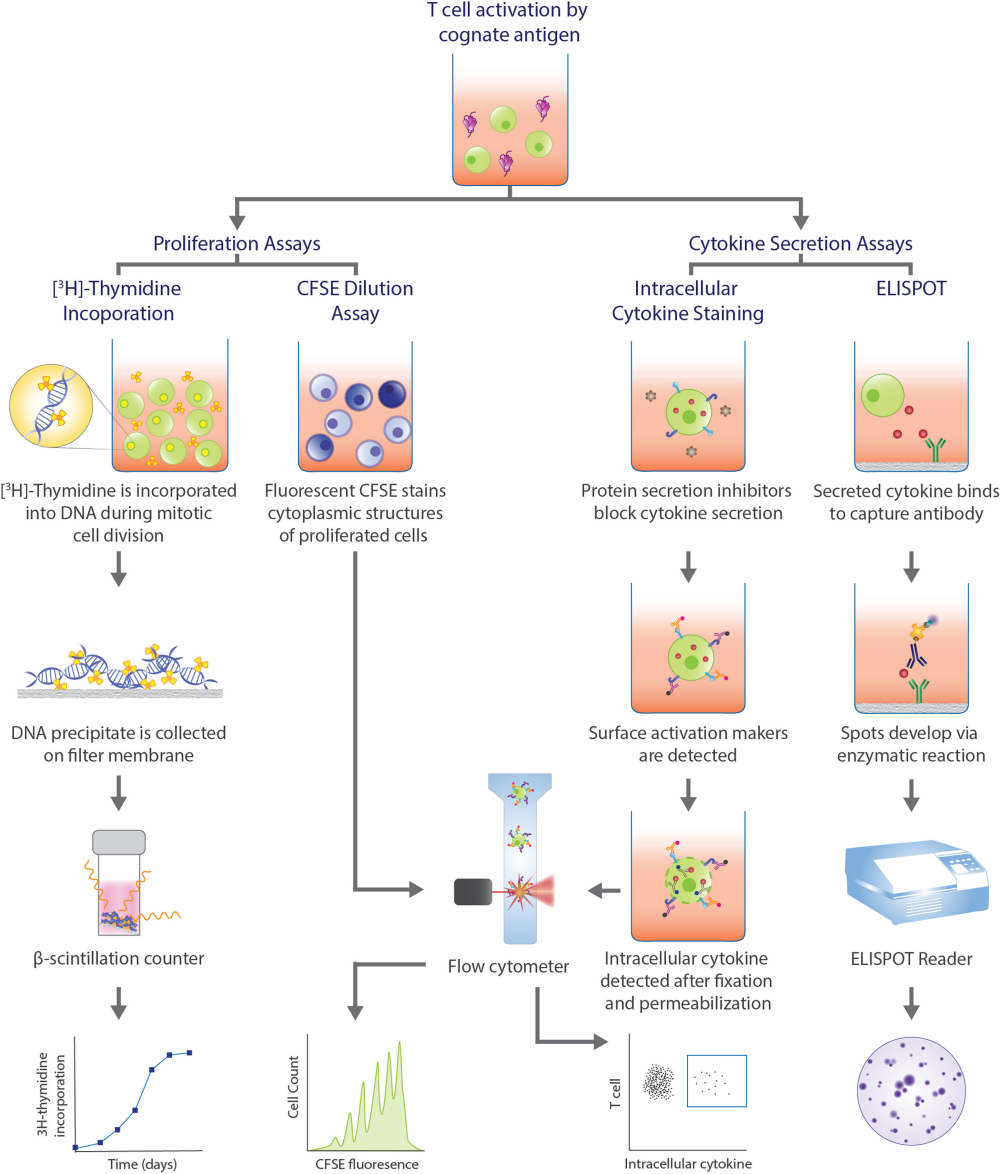
Functional assays commonly used in human T cell studies. Functional assays can be categorized into those that assess proliferative capacity of antigen-specific T cells and assays that analyze cytokine profiles upon T cell recognition of cognate antigen and subsequent activation. Proliferation assays can measure the amount of radioactive [^3^H]-thymidine incorporated into the DNA during cell division, with greater radioactivity indicating greater cell division. Alternatively, the level of fluorescence emitted by cells stained with carboxyfluorescein succinimidyl ester (CFSE) can be detected by flow cytometry, with greater number divisions correlating with lower fluorescence. In intracellular cytokine staining (ICS), protein secretion inhibitors, such as brefeldin A or monensin, allows for examination of cytokine production within a cell. Staining surface activation markers allows for phenotyping. Following fixation and permeabilization, the trapped intracellular cytokines are stained with fluorescent antibodies which can be detected *via* flow cytometry. Enzyme-linked immunospot (ELISPOT) is a popular method to assess cytokine secretion. The cytokine of interest secreted from an activated T cell is bound to a capture antibody on a PVDF bottom well. A biotinylated detection antibody also binds to the cytokine and facilitates the interaction between streptavidin-conjugated enzyme and its substrate to produce a color spot. Spots are quantified with a ELISPOT plate reader. Each spot represents one reactive cell.

Detection of antigen-specific T cells is a vital step, but determining the functional capacity of the identified cells in producing a robust immune response is equally important and relies on functional assays (Figure 2). Methods measuring [^3^H]-thymidine incorporation into lymphocyte DNA (246) and dilution of carboxyfluorescein succinimidyl ester (CFSE) dye bound to amine groups of intracellular molecules (247) during cell division have been a mainstay for evaluating the proliferation of lymphocytes in response to antigens. These procedures circumvent the issue of low frequency of target cells. However, as it is often PBMCs that are cultured, and not sorted T cells, there is the possibility of bystander activation, which decreases the specificity of the response observed and the frequency of the antigen-specific T cells cannot be accurately extrapolated. In the case of [^3^H]-thymidine incorporation assay, the proliferative cell subpopulation cannot be phenotyped, whereas with CFSE dilution assay, cell subpopulations may be delineated with surface markers and flow cytometry analysis. However, the dye can interfere with expression of activation markers (248).

Functional assays that examine cytokine production allows for classification of the cells into different subsets that are distinguished by different effector functions. This is particularly valuable for CD4^+^ T cells that can be categorized as Th1, Th2, and Th17 cells, for example. Two classic procedures that assess cytokine production are enzyme-linked immunospot (ELISPOT) and intracellular cytokine staining (ICS) (Figure 2). While an ELISPOT detects secreted cytokines induced by antigens, ICS reveals cytokine production within the golgi/ER bodies upon permeabilization of the cell and treatment with protein secretion inhibitors, such as brefeldin A or monensin. Both techniques are sensitive for detection of antigen-specific T cells and allow for enumeration and characterization at a single-cell level. However, as both techniques also depend on postulating cytokines relevant for the disease, it is possible that the frequency of cytokine-producing cells is underestimated if cells secrete cytokines other than the one of interest. Additionally, the breadth of cytokine analysis is limited in an ELISPOT to only two cytokines for a given experiment (249). The source of the cytokine cannot be determined with an ELISPOT, making it an indirect method of T cell identification. Conversely, ICS allows for the simultaneous detection of cytokines and the phenotype of the cytokine-producing cells by the addition of activation marker fluorescent antibodies. Hence, pairing with pMHC multimer or activation marker staining enhances the specificity of the reaction observed and provides a complete assessment of the antigen-specific response.

Pre-enrichment is a modification often made to the above methods to overcome the issue of low target cells and further improve the sensitivity offered by flow cytometry. A common approach is *in vitro* expansion, wherein PBMCs are cultured in the presence of the antigen over one to two weeks to preferentially grow antigen-specific T cells. However, similar to proliferation assays, a drawback of this procedure is the risk of activating non-T cells present in the sample, thereby increasing background and reducing the accuracy of the quoted frequency of antigen-specific T cells (236). Alternatively, enriching the target cell population *via* magnetic separation greatly increases sensitivity and provides insight into previously unidentified T cell subpopulations, provided that highly specific markers were utilized, whether that be pMHC multimers or activation markers (236, 238).

Employing the right technique may lead to discovery of T cells in disease. Each method explores different aspects of T cell biology. Taking this into consideration and the rarity of antigen-specific T cells, it is advantageous to integrate the various approaches for a more reliable and holistic understanding of the cellular mechanisms at play.

## Future Directions

Cellular immunity is a key player in the autoimmune response, as evidenced by the growing number of studies in both T cell-mediated and antibody-associated CNS disorders. Yet, there is much to be learnt of T cell contribution to the complexities of CNS autoimmunity. Investigating the underlying cellular mechanisms can deepen our understanding of disease pathogenesis, especially in the expanding range of neurological diseases recently associated with antibodies, in patients seronegative for antibodies but suspected to have immune dysregulation, in differentiating clinically similar diseases with heterogeneous pathology, and in conditions currently classified as idiopathic. Importantly, this new found knowledge can lead to the development of improved diagnostic tools and also translate into novel immunotherapeutics that are more targeted against T cells or their cytokines, like tocilizumab and IL-17-directed secukinumab (250), which can be more effective than the current treatment regime given the unique environment of the CNS.

## Author Contributions

DP and AZ performed the literature review, wrote the manuscript, and contributed equally to the work. DP and FT designed the figures. FB conceived the concept, critically revised, and oversaw the process of manuscript preparation. All authors read, edited, and approved the final manuscript.

## Conflict of Interest Statement

The authors declare that the research was conducted in the absence of any commercial or financial relationships that could be construed as a potential conflict of interest.
